# Targeting Neutrophilic Inflammation Using Polymersome-Mediated Cellular Delivery

**DOI:** 10.4049/jimmunol.1601901

**Published:** 2017-03-13

**Authors:** James D. Robertson, Jon R. Ward, Milagros Avila-Olias, Giuseppe Battaglia, Stephen A. Renshaw

**Affiliations:** * Department of Biomedical Science, University College London, London WC1E 6BT, United Kingdom;; † Division of Infection and Immunity, University College London, London WC1E 6BT, United Kingdom;; ‡ The Bateson Centre, The University of Sheffield, Western Bank, Sheffield S10 2TN, United Kingdom;; § Department of Chemistry, University College London, London WC1E 6BT, United Kingdom; and; ¶ The Medical Research Council/University College London Centre for Molecular and Medical Virology, University College London, London WC1E 6BT, United Kingdom

## Abstract

Neutrophils are key effector cells in inflammation and play an important role in neutralizing invading pathogens. During inflammation resolution, neutrophils undergo apoptosis before they are removed by macrophages, but if apoptosis is delayed, neutrophils can cause extensive tissue damage and chronic disease. Promotion of neutrophil apoptosis is a potential therapeutic approach for treating persistent inflammation, yet neutrophils have proven difficult cells to manipulate experimentally. In this study, we deliver therapeutic compounds to neutrophils using biocompatible, nanometer-sized synthetic vesicles, or polymersomes, which are internalized by binding to scavenger receptors and subsequently escape the early endosome through a pH-triggered disassembly mechanism. This allows polymersomes to deliver molecules into the cell cytosol of neutrophils without causing cellular activation. After optimizing polymersome size, we show that polymersomes can deliver the cyclin-dependent kinase inhibitor (R)-roscovitine into human neutrophils to promote apoptosis in vitro. Finally, using a transgenic zebrafish model, we show that encapsulated (R)-roscovitine can speed up inflammation resolution in vivo more efficiently than the free drug. These results show that polymersomes are effective intracellular carriers for drug delivery into neutrophils. This has important consequences for the study of neutrophil biology and the development of neutrophil-targeted therapeutics.

## Introduction

Neutrophils are the most abundant leukocyte in mammals and are the first cells recruited to sites of infection. The main role of neutrophils is to remove microorganisms through phagocytosis (1). Internalized microorganisms are rapidly degraded in the phagosome using a destructive mixture of proteases, antimicrobial peptides, and reactive oxygen species, which are delivered by cytoplasmic granules (2). Killing pathogens intracellularly allows neutrophils to remove microorganisms without causing off-target damage to the host; however, not all microorganisms can be removed by this method, particularly if contact is obstructed by the extracellular matrix (3). Following activation, neutrophils degranulate, expelling their destructive contents into the extracellular environment (4). This degranulation helps ensure that the surrounding area is sterilized, and collapses nearby capillaries and lymphatic vessels to prevent microorganisms from escaping (3). Yet, unsurprisingly, degranulation can cause extensive tissue damage, so this process must be tightly controlled. Therefore, once an infection has been cleared, neutrophil apoptosis is an important step in preventing further damage to the host (5).

Neutrophil apoptosis can be initiated following phagocytosis of target pathogens, or, in the absence of survival signals, it can occur spontaneously (6). This ensures that neutrophils cease proinflammatory functions and allows their toxic contents to be packaged within apoptotic bodies for safe clearance by macrophages. Upregulation of phosphatidylserine and other “eat-me” signals during apoptosis help ensure that neutrophil clearance occurs before the contents are lost through secondary necrosis (7).

Not only does apoptosis prevent neutrophils from responding to proinflammatory stimuli, but apoptotic neutrophils can also actively promote inflammation resolution by sequestering cytokines (8) and converting macrophages to a proresolution phenotype following efferocytosis (9). However, apoptosis can be delayed by stimulation with survival factors such as GM-CSF, with prolonged neutrophil survival leading to excessive tissue damage, as observed in multiple inflammatory diseases (10–12).

Driving neutrophil apoptosis is a potential therapeutic strategy for the treatment of inflammatory diseases. This can be achieved by drug therapy with Tanshinone IIA or cyclin-dependent kinase inhibitors (CDKi) (13). The broad spectrum CDKi (R)-roscovitine, for instance, has emerged as a potent inducer of neutrophil apoptosis (14–16) even in the presence of survival factors such as GM-CSF (17). Drug delivery carriers may enable more efficient access to the neutrophil intracellular milieu to help encourage drug signaling processes and limit off-target side effects. However, cellular delivery and genetic manipulation of neutrophils has proven difficult. This has not only prevented us from manipulating neutrophils for therapeutic intervention, but has also limited our understanding of the molecular pathways that control neutrophil function. A number of neutrophil transfection techniques have been attempted, but none have become well established (18–21).

In this study PMPC-PDPA polymersomes are explored as drug delivery vectors for neutrophils. Polymersomes were tested for adverse effects on neutrophil viability and IL-8 release, and their size was optimized for efficient cargo delivery. Finally, the ability of polymersomes to encapsulate and deliver (R)-roscovitine into neutrophils was tested in vitro and in a zebrafish model of neutrophilic inflammation.

## Materials and Methods

### Reagents

Unless otherwise stated, reagents were purchased from Sigma-Aldrich. Chloroform and methanol were purchased from Fisher Scientific. (R)-roscovitine was purchased from Cambridge Bioscience, GM-CSF from PeproTech, and cascade blue from Life Technologies. The IL-8 ELISA kit was purchased from R&D Systems.

### Polymersome formation

Polymersomes were assembled using the pH-switch or film-rehydration method as described previously (22). The synthesis of PMPC_25_-PDPA_65_ and rhodamine 6G-labeled PMPC_25_-PDPA_70_ was performed by reversible addition fragmentation chain-transfer polymerization, and atom-transfer radical polymerization respectively (23, 24). Polymersomes were purified using crossflow filtration and differential centrifugation as described previously (25). Briefly, polymersomes were purified into different sizes by initially extracting the smallest nanoparticles using a KrosFlo Research II*i* System with a 50 nm hollow fiber filter module, all purchased from Spectrum Laboratories. This was followed by differential centrifugation at increasingly higher centrifuge speeds (25).

### Polymersome uptake by human neutrophils

Peripheral blood was extracted from healthy donors as approved by the South Sheffield Research Ethics Committee (STH13927). Neutrophils were purified from the peripheral blood of healthy donors using a Percoll density gradient as described previously, typically yielding neutrophil purities of >95% (26, 27). For cytospin apoptosis counts and cytokine ELISAs, neutrophils were further purified using negative magnetic selection, to ensure that contaminating PMBCs did not influence neutrophil responses (28, 29). All other experiments on human neutrophils were using neutrophils purified using the Percoll gradient only. In the initial neutrophil viability and IL-8 ELISAs, polymersomes were not purified to a specific size fraction. Viability was assessed either by observation of nuclear morphology on histochemically stained cytospin preparations according to well-accepted protocols (30), or by flow cytometry of neutrophils stained with Alexa Fluor 647 annexin V and propidium iodide (BioLegend). Flow cytometry was performed with a BD FACSArray Bioanalyzer with 10,000 events captured within the neutrophil gate. Data were analyzed using FlowJo software (Tree Star, OR). This experiment was repeated with a similar protocol on FaDus purchased from the American Type Culture Collection (Manassas, VA), seeded in 24-well plates, and grown for 1 d in supplemented DMEM.

Cytokine release was measured using an IL-8 ELISA using matched Ab pairs at optimized concentrations as described previously (31). To assess the delivery of polymersomes encapsulating rhodamine B octadecyl ester perchlorate (rhodamine B), neutrophils were incubated with 1 mg/ml of CelLuminate for 6 or 24 h, and then washed, fixed with 1× Cellfix (Becton Dickinson) and visualized on a Perkin-Elmer UltraVIEW VoX spinning disk confocal microscope with a 514 nm laser and a 40× oil immersion lens.

### Optimization of polymersome size

Purified polymersome size fractions were incubated with neutrophils at 37°C for the desired time period. Neutrophils were centrifuged at 300 RCF for 3 min with a table-top centrifuge (Eppendorf Minispin) and the pellet was then washed with ice-cold PBS; this washing step was repeated. Neutrophils were kept on ice before the fluorescence intensity was measured using an LSR II Flow Cytometer (BD Biosciences) with 10,000 events captured within the neutrophil gate and a 450 nm violet laser (cascade blue) or a 575 nm blue laser (Rho-PMPC-PDPA). The relative median fluorescence intensity (rMFI) was obtained by dividing the MFI of the treated neutrophils by the MFI of the untreated neutrophils and subtracting one, so that an MFI value equal to the control had an rMFI of 0. Microscopy was undertaken on cells fixed with 1× Cellfix (Becton Dickinson) and visualized with a Perkin-Elmer UltraVIEW VoX spinning disk confocal or an Olympus FV1000 confocal microscope.

### Calculation of encapsulated (R)-roscovitine concentration

Following (R)-roscovitine encapsulation, free drug was removed using gel permeation chromatography (32) and the polymersomes were purified to the desired size as described above. To measure the concentration of polymer and encapsulated (R)-roscovitine, reverse-phase HPLC (RP-HPLC) was performed on a Dionex Ultimate 3000 system with an associated ultraviolet-Vis spectrophotometer. The sample pH was lowered to six using 1 M HCL to disassemble the polymersomes. The samples were then passed through a C18 analytical column (Phenomenex Jupiter; 300 Å, 150 × 4.6 mm, 5 μm) and run in a multistep gradient of eluent a: methanol + 0.1% trifluoroacetic acid, and eluent b: H_2_O + 0.1% trifluoroacetic acid. The gradient was as follows: 0 min 5%, 6.5 min 5%, 12 min 100%, 14 min 100%, 15 min 5%, 20 min 5%. RP-HPLC ultraviolet-Vis calibration curves were used to calculate the concentration of polymer and encapsulated (R)-roscovitine.

### Zebrafish husbandry and assays

Zebrafish were raised and maintained according to standard protocols (33) in U.K. Home Office-approved aquaria at the University of Sheffield Zebrafish Facility in the Bateson Centre. The transgenic *Tg*(*mpx:GFP*)*i114* line (*mpx:*GFP), was used in both zebrafish experiments. Injury by tail fin transection was performed as previously described (34). Three days postfertilization (dpf) *mpx:*GFP zebrafish embryos were immersed in 0.02% 3‐amino benzoic acid ethyl ester (tricaine), then placed on masking tape on a Petri dish lid. The embryos were injured by complete tail fin transection at the caudal fin with a microscalpel (World Precision Instruments). Four hours following injury, zebrafish were visualized under a fluorescent dissecting microscope (Leica MZ10F) and embryos with 20–25 neutrophils at the injury site (posterior to the circulatory loop) were selected, transferred to a 96-well plate and polymersomes or their controls were added to the zebrafish media. Zebrafish were then washed and visualized using a Perkin-Elmer UltraVIEW VoX spinning disk confocal microscope or the number of neutrophils at the injury site was counted under a fluorescent dissecting microscope.

### Statistical analysis

Data were analyzed using a two-way ANOVA or a one-way ANOVA with Bonferroni posttest (Prism 5.0; GraphPad Software).

## Results

### Polymersomes are biocompatible intracellular vectors for human neutrophils

In a similar manner to membrane phospholipids, the amphiphilic PMPC-PDPA copolymer contains a hydrophilic group (PMPC) and a hydrophobic group (PDPA), which enables the copolymer to self-assemble into a membrane in aqueous solutions (35). We have shown that these membranes wrap into vesicles, otherwise known as polymersomes, which are capable of encapsulating a variety of molecules, proteins, and nucleic acids (36–38). The DPA component of this copolymer is a weak base with a pK_a_ of 6.4 under physiological conditions. Once internalized, polymersomes are transported into early endosomes where the drop in pH triggers polymersome disassembly, resulting in a sudden increase in the number of charged molecules (39). This leads to an osmotic shock, triggering temporary destabilization of the endosome and release of some of the polymersome cargo into the cell cytosol (38). Importantly, the pK_a_ of the DPA group ensures this process occurs very early on in the endolysosomal pathway, enabling release of the polymersome cargo without provoking cellular toxicity (39).

The PMPC block is a biocompatible polymer that is highly resistant to protein adhesion, and widely used as a coating for contact lenses, coronary stents, and artificial joints (40–42). Although PMPC-PDPA are resistant to unspecific protein adhesion, we have shown previously that the phosphorylcholine groups have high affinity for scavenger receptor B1 enabling them to bind and become internalized by a variety of cell types, particularly immune cells and cancer cells, which are rich in scavenger receptors (39, 43).

**FIGURE 1.**
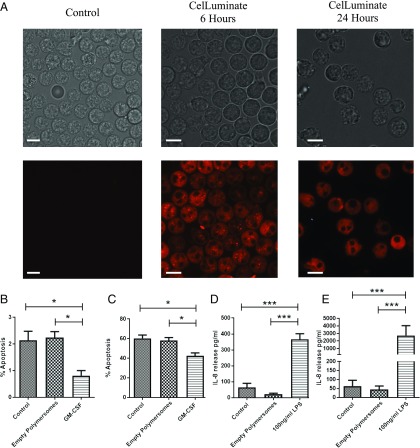
Uptake of polymersomes by human neutrophils. (**A**) Confocal photomicrograph of neutrophils incubated with polymersomes encapsulating rhodamine B octadecyl ester perchlorate for 6 or 24 h at a final concentration of 1 mg/ml, scale bar, 8 μm. (**B**) Apoptosis assays on primary human neutrophils after incubation with empty polymersomes or GM-CSF (0.01 μg/ml) for 4 h or (**C**) 20 h. (**D**) IL-8 levels assayed by ELISA on human neutrophils after incubation with empty polymersomes or controls for 4 h or (**E**) 6 h. Neutrophils treated with polymersomes were not significantly different from the negative control group in all experiments (one-way ANOVA with a Bonferroni multiple comparison test, error bars = SEM from three independent experiments, *p < 0.05, ***p < 0.001).

To explore the potential of PMPC-PDPA polymersomes as intracellular delivery vectors for neutrophils, human peripheral blood neutrophils were purified from the blood of healthy donors using a Percoll density gradient and the cells were incubated with polymersomes encapsulating the fluorescent dye rhodamine B. After 6 or 24 h incubation, the cells were washed then examined by confocal microscopy, revealing delivered rhodamine was dispersed throughout the interior of the cell (Fig. 1A, Supplemental Video 1).

Extensive toxicity analysis on over 20 different cell types has demonstrated that PMPC-PDPA polymersomes are well tolerated, even at relatively high concentrations (39). However, neutrophils are very sensitive cells and many manipulations cause neutrophil activation or cell death (26).

To ensure that polymersomes alone do not alter neutrophil life span, the rate of neutrophil apoptosis after incubation with 1 mg/ml of polymersomes for 4 or 20 h was measured by observation of nuclear morphology on histochemically stained cytospin preparations according to well-accepted protocols (30). Treatment with GM-CSF (0.01 μg/ml) resulted in a reduction in neutrophil apoptosis at both time points, but empty polymersomes had no effect on neutrophil apoptosis counts compared with the PBS control (Fig. 1B, 1C).

Activation of neutrophils and initiation of their proinflammatory response results in the production and release of inflammatory cytokines such as IL-8 (44). IL-8 is a potent chemokine that is involved in recruiting neutrophils and coordinating the immune response. To determine the effect of polymersomes on neutrophil activation, neutrophils were incubated with polymersomes for 4 or 6 h. Supernatants were extracted and IL-8 concentration was measured by ELISA. Incubation with polymersomes at a concentration of 1 mg/ml had no effect on neutrophil IL-8 release compared with the PBS control (Fig. 1D, 1E). In contrast, the positive control (LPS, LPS) activated neutrophils and increased the amount of IL-8 release at both time points.

### Optimizing polymersome size for efficient cargo delivery

Nanoparticle size is known to have a critical effect on numerous aspects of the nanoparticle in vivo function including: circulation time, cell binding, internalization, extravasation, and biodistribution (45–47). Controlling the rate of internalization will influence both the efficiency and specificity of a drug vector for a specific cell type. However, the rate of internalization can be difficult to predict theoretically because it is also dependent on nanoparticle surface chemistry (39), ligand surface density (48), receptor expression and binding affinity (49), the mechanism(s) of receptor-mediated endocytosis (50), and even the degree of nanoparticle interactions on the plasma membrane (51). Thus, the optimal nanoparticle size depends on the specific surface properties of the nanoparticle and the target cell (45), and must be determined experimentally.

Following endosomal release, polymersome-forming copolymer chains integrate with cell membranes (39). As shown in Supplemental Fig. 1A, neutrophils incubated with rhodamine-labeled polymersomes (where rhodamine is covently bound to individual copolymer chains) show a strong signal from membranous compartments (Supplemental Fig. 1A). The aminocoumarin fluorophore cascade blue is a water-soluble, cell-impermeable fluorescent dye often used to measure membrane permeability (52). When polymersomes loaded with cascade blue were incubated with neutrophils, the signal was distributed throughout the cell cytosol (Supplemental Fig. 1A). To investigate the consequence of polymersome size on internalization by human neutrophils, rhodamine-labeled polymersomes encapsulating cascade blue were formed by the pH-switch method. By encapsulating cascade blue in rhodamine-labeled polymersomes, it was possible to track the amount of polymer internalized by the cell, as well as the amount of delivered cargo.

Rhodamine-labeled polymersomes encapsulating cascade blue were separated into six size fractions using crossflow filtration followed by differential centrifugation as described previously (25). The concentration of cascade blue and rhodamine-labeled polymer was measured using fluorescence spectroscopy. Human neutrophils were incubated with one of the six purified polymersome size fractions at a final polymer concentration of 0.1 mg/ml. At multiple time points throughout the experiment, neutrophils were analyzed by flow cytometry. Example dot plots are shown in Supplemental Fig. 1B.

**FIGURE 2.**
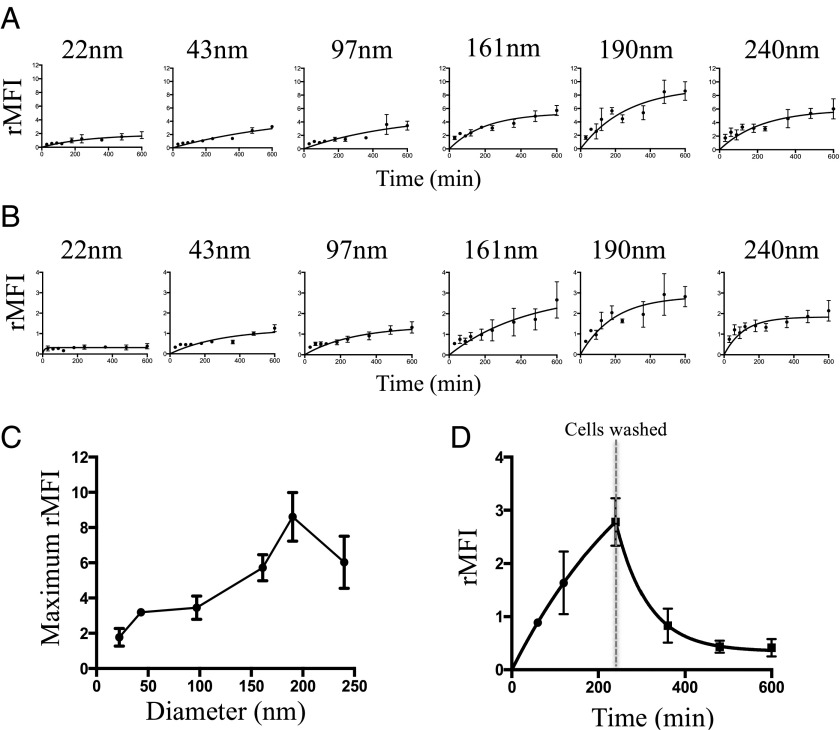
Quantified neutrophil rMFI over time after incubation with rhodamine-labeled polymersomes encapsulating cascade blue. (**A**) Neutrophil rMFI in the rhodamine channel showing the amount of rhodamine-labeled polymer internalized over time for each of the size fractions. (**B**) Neutrophil rMFI in the cascade blue channel showing the amount of cascade blue delivered into neutrophils over time for each size fraction. The graphs show that neutrophil fluorescence from the polymer and cascade blue increases over time with a plateau at later time points. The extent of neutrophil fluorescence was dependent on the polymersome size. (**C**) Neutrophil rMFI after incubation with polymersomes at each size fraction for 600 min. (**D**) rMFI of neutrophils treated with rhodamine-labeled polymersomes and washed after 240 min to determine the rate of polymer release (on all graphs error bars = SEM, from three independent experiments).

Incubation with polymersomes resulted in a rapid increase in the normalized neutrophil fluorescence intensity, referred to as the rMFI. The degree of neutrophil fluorescence over time was dependent on the polymersome size (Fig. 2A, 2B). Neutrophils treated with the 190 nm polymersome fraction appeared to have the highest internalization of rhodamine labeled polymer and cascade blue. By plotting the data from the final time point only, it can be seen that the amount of polymer internalized by neutrophils escalates with increasing diameters up to 190 nm (Fig. 2C). This is consistent with previous reports showing that spherical nanoparticle uptake is optimal at a given critical radius, which depends on the receptor/ligand affinity and receptor density (53).

To investigate the rate at which the polymersome signal drops from neutrophils following internalization, neutrophils were incubated with 190 nm polymersomes using the same protocol. After 240 min the neutrophils were pelleted by centrifugation, washed, and returned to normal media. The neutrophil rMFI was measured at three further time points to determine the rate of polymer release from the cells. Interestingly, once the extracellular polymersomes were removed, neutrophil rMFI rapidly decreased (Fig. 2D). This may be due to polymer metabolism or secretion.

The uptake of polymersomes in vivo will depend not only on the rate of internalization by neutrophils at a given nanoparticle size, but also the relative rate of internalization by other cell types. Therefore, the size-dependent internalization experiment was repeated using the cell line FaDu for comparison. Once again, FaDu cells were incubated with one of six purified polymersome size fractions and analyzed by flow cytometry at multiple time points up to 25 h. In agreement with the complementary neutrophil experiment, the fluorescence from both the cascade blue channel and the rhodamine channel increased over time and then plateaued (Supplemental Fig. 2A, 2B). However, the effect of size on internalization was far less marked (Supplemental Fig. 2C).

### Encapsulated (R)-roscovitine promotes neutrophil apoptosis

The broad spectrum CDKi (R)-roscovitine drives neutrophil apoptosis by downregulating the survival protein Mcl-1 (14); however, clinical use may be restricted due to off-target effects on non-immune cell populations. To overcome this potential limitation, (R)-roscovitine was encapsulated within polymersomes using the film rehydration method (54). Following (R)-roscovitine encapsulation, polymersomes were purified to a mean diameter of ∼200 nm (Supplemental Fig. 3A). Encapsulation of (R)-roscovitine was calculated by RP-HPLC associated with ultraviolet detection (Supplemental Fig. 3B, 3C).

**FIGURE 3.**
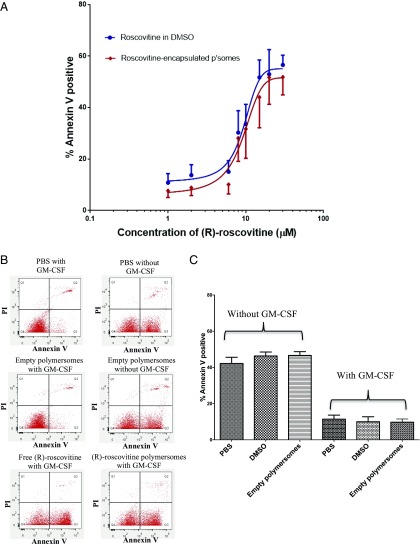
Encapsulated and free (R)-roscovitine promote neutrophil apoptosis. (**A**) Dose response line graph showing the percentage of apoptotic neutrophils (annexin V positive) when incubated with increasing concentrations of encapsulated or free (R)-roscovitine for 8 h. Treatment with roscovitine significantly increased neutrophil apoptosis in both groups but encapsulation within polymersomes did not significantly affect apoptosis rates compared with free roscovitine (two-way ANOVA, error bars show SEM from four independent experiments, *p* < 0.0001). (**B**) Dot plots from flow cytometry of human neutrophils showing annexin V and propidium iodide staining after 8 h incubation with treatment or controls (GM-CSF at 0.01 μg/ml, (R)-roscovitine at 30 μM, and PMPC-PDPA polymer at 1.15 mg/ml). (**C**) Bar graph showing the percentage of apoptotic neutrophils (annexin V positive) after 8 h incubation with PBS, DMSO, or empty polymersomes. Treatment with empty polymersomes did not significantly alter the rate of apoptosis (GM-CSF at 0.01 μg/ml, PMPC-PDPA polymer at 1.15 mg/ml) (one-way ANOVA, error bars = SEM from four independent experiments).

Human neutrophils were incubated for 8 h with GM-CSF (0.01 μg/ml) and 1–30 μM of encapsulated (R)-roscovitine or free (R)-roscovitine. Cell viability was measured by flow cytometry with annexin V and propidium iodide staining. Incubation of polymersomes with encapsulated or free (R)-roscovitine resulted in a dose-dependent increase in neutrophil apoptosis, but the rate of apoptosis was not significantly different between these groups (Fig. 3A). Example dot plots in Fig. 3B demonstrate similar levels of annexin V–positive and propidium iodide–positive cells were observed in neutrophils treated with polymersomes compared with their controls. Empty polymersomes had no effect on neutrophil viability compared with cells treated with PBS alone (Fig. 3C).

### Encapsulated (R)-roscovitine promotes inflammation resolution in an in vivo Zebrafish model

Previous studies in our laboratory have shown that polymersomes can improve the delivery of molecules into tissues due to their intrinsic flexibility (55, 56). For instance, PMPC-PDPA polymersomes have enabled the delivery of fluorescent dextran and large Igs into ex vivo human skin, the delivery of antibiotics into human mucosa, and the delivery of small molecules into the central core of a multicellular tumor spheroid (57, 58). Enhanced delivery of (R)-roscovitine through the skin may help in the treatment of inflammatory skin diseases such as psoriasis or severe eczema.

**FIGURE 4.**
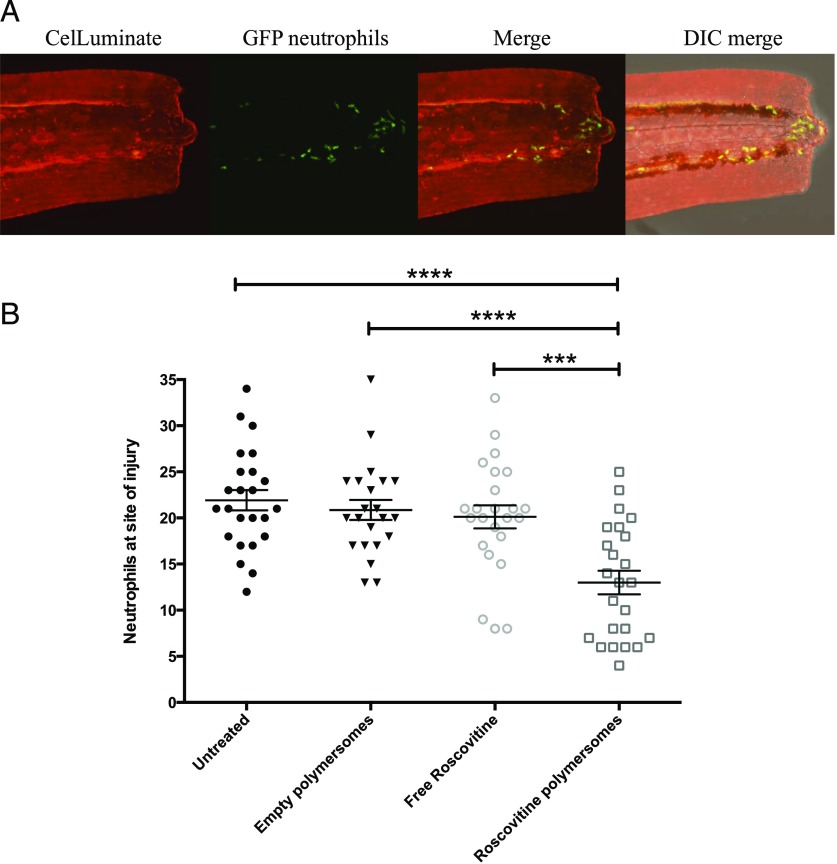
Polymersome-mediated delivery of (R)-roscovitine into the zebrafish tail speeds up inflammation resolution. Zebrafish embryos (*mpx*:GFP) 3 dpf were subject to tail injury by transection of the tail end. (**A**) At 4 hpi zebrafish embryos were incubated with polymersomes encapsulating rhodamine B octadecyl ester perchlorate (CelLuminate) for 8 h before the fish were washed and imaged by confocal microscopy. Original magnification ×10. (**B**) Zebrafish embryos were treated with 30 μM free or encapsulated (R)-roscovitine at 4 hpi. At 12 hpi neutrophils numbers at the injury site were counted using a dissecting microscope. Encapsulated (R)-roscovitine significantly improved inflammation resolution compared with free roscovitine or controls (one-way ANOVA with Bonferroni post hoc comparison, lines represent the mean ± SEM from three independent experiments, ***p < 0.001, ****p < 0.0001).

To study the potential benefits of polymersomes in the treatment of neutrophil-dominated inflammatory diseases, Zebrafish were employed as an animal model. Zebrafish provide an excellent model for studying the innate immune system because the embryos are transparent and contain neutrophils that can be fluorescently labeled by transgenesis. Zebrafish neutrophils share morphological, biochemical, and functional similarity with human neutrophils and we have shown they respond in a similar manner to chemicals known to influence neutrophil function (13, 59). Zebrafish tail fin transection results in sterile inflammation characterized by neutrophil recruitment peaking at 6 h postinjury (hpi) and resolution of neutrophilic inflammation by 24 hpi (33). This provides a relatively cheap, rapid, and effective method for testing the potency of anti-inflammatory compounds in promoting inflammation resolution (13, 59). The transgenic *Tg*(*mpx:GFP*)*i114* line specifically labels neutrophils with GFP, which allows neutrophils to be tracked in vivo using a fluorescence microscope (33). To study polymersome delivery in vivo, 3 dpf *mpx:*GFP zebrafish embryos were subject to tail injury by transection of the tail end and incubated with polymersomes encapsulating rhodamine B for 8 h. Visualization of the tail region by confocal microscopy revealed a diffuse rhodamine signal throughout the tail (Fig. 4A).

To compare the ability of encapsulated and free (R)-roscovitine to promote inflammation resolution in vivo, *mpx:*GFP zebrafish were injured by tail fin transection and treated at 4 hpi with 30 μM free (R)-roscovitine, 30 μM of polymersome-encapsulated (R)-roscovitine, or their respective controls. At 12 hpi the neutrophils at the site of injury were counted under a fluorescence microscope. As shown in Fig. 4B, polymersome-encapsulated (R)-roscovitine significantly reduced the number of neutrophils at the site of injury at 12 hpi compared with free (R)-roscovitine or control. These results demonstrate that (R)-roscovitine encapsulated in polymersomes can speed up inflammation resolution in vivo significantly better than the free drug.

## Discussion

Neutrophils have proven very difficult cells to manipulate, and to our knowledge no commercially available vector exists that enables the efficient intracellular delivery of cargo within their short life span without compromising their viability and activation state. The ability of PMPC-PDPA polymersomes to deliver cargo into neutrophils without detrimental effects may be attributed to a number of features. It is important to emphasize that most transfection agents are based on cationic lipids or polymers forming positively charged nanoparticles that interact strongly with the negatively charged cell membrane favoring binding and delivery (60). This strong interaction, however, leads to unspecific binding to TLR, such as TLR4, with consequent activation of leukocytes (61). Therefore, it is not surprising that lipoplex, the most-studied non-viral nucleic acid vector, causes systemic inflammation and toxicity when injected into mice (62, 63). Conversely, the neutral charge of the polymersome membrane prevents non-specific interactions between polymersomes and proteins. This property, known as anti-fouling, is crucial for translation of synthetic polymers into the clinic and is responsible for the proven non-immunogenicity of PMPC (41).

It is likely that the rapid internalization of polymersomes is mediated through a high binding affinity for their target receptors. It has previously been shown that the class B scavenger receptors, scavenger receptors B1 and B2 (SR-BI/SRBII), and CD36 are essential for the internalization of PMPC-PDPA polymersomes into a number of cell types (43). Indeed, CD36 and SR-BI/SRBII are both expressed by neutrophils, which may explain the fast rate of polymersome internalization (64, 65).

The accumulation of polymersomes within neutrophils will depend on both the rate of internalization and the rate of removal. Incubation of human neutrophils with polymersomes resulted in an increase in fluorescence followed by a gradual plateau. Interestingly, when the polymersomes were removed from solution, the fluorescence intensity rapidly declined. This indicates that the polymer is either degraded or released from the cells over time. This may also explain the gradual plateau in fluorescence due to a balance in polymersome internalization and release. Rapid release of polymersomes following cargo delivery may be beneficial, preventing the unnecessary intracellular accumulation of polymer within the cells, but further work will be necessary to confirm this.

The rate of nanoparticle internalization is dependent on a number of factors. Internalization can be driven in part simply from the physical interaction of a nanoparticle with the cell membrane. By modeling membrane wrapping, the Freund group calculated that there is an optimal nanoparticle diameter of ∼50–60 nm, which facilitates membrane wrapping in the shortest time period (49). However, the rate of internalization experimentally varies depending on the binding affinity and the properties of the nanoparticle and its target cell (66–70).

In this study, polymersomes with a diameter of ∼190 nm resulted in the greatest increase in neutrophil fluorescence intensity both when considering the fluorescence from the rhodamine-labeled polymer and encapsulated cascade blue. It should be emphasized that the increase in neutrophil fluorescence intensity from the rhodamine demonstrates that 190 nm polymersomes are most efficient in terms of the amount of polymer delivered into the cells, but not the number of polymersomes internalized. This is because the larger polymersomes are formed from a greater amount of polymer, and hence more rhodamine is delivered into the neutrophil for every polymersome that is internalized. However, the data highlight that this size of polymersome may be optimal as a drug-delivery vehicle for neutrophils.

The rate of polymersome internalization into FaDu cells compared with neutrophils cannot be compared by fluorescence intensity alone. However, these data show that FaDu cells, unlike neutrophils, appeared to internalize polymersomes at a similar rate at all sizes tested. The reason for this is unclear, but may be a result of the different density of receptors on their cell membrane. Alternatively, the specialized abilities of neutrophils to internalize larger foreign particles may improve their uptake of polymersomes with larger diameters. These data suggest that size will influence the amount of polymersomes that are internalized into neutrophils compared with other cells. When targeting neutrophils, polymersomes with a diameter of ∼200 nm may be effective, whereas a smaller diameter of ∼50 nm may allow a greater degree of internalization by cancer cells. (R)-roscovitine encapsulated in polymersomes promoted neutrophil apoptosis in vitro in a dose-dependent manner. The ability of encapsulated (R)-roscovitine to promote neutrophil apoptosis was not significantly different to that of free (R)-roscovitine. This suggests the mass of (R)-roscovitine delivered into neutrophils using polymersomes is similar to the mass of free (R)-roscovitine that enters cells by other means.

Zebrafish provide an excellent model for studying the innate immune system. The transparent embryos contain neutrophils and macrophages, which can be fluorescently labeled by transgenesis, and by 3 dpf the innate immune system is fully functional, allowing its role in infection and inflammation to be understood in the absence of the adaptive immune system that does not fully mature until 1 mo postfertilization (71). The zebrafish has been helpful in the study of neutrophil biology, leading to a number of discoveries in the field, including the role of neutrophil reverse migration in inflammation resolution (34, 72, 73). Using the zebrafish tail transection model, it was shown that (R)-roscovitine encapsulated within polymersomes promoted inflammation resolution much more quickly than the free drug. Free (R)-roscovitine alone did not significantly reduce neutrophil numbers at the injury site compared with the untreated control, although the mean number of neutrophils was slightly lower (not significant). The improvement of encapsulated (R)-roscovitine compared with the free drug suggests that polymersomes were more efficient at delivering the drug into zebrafish neutrophils than could be achieved by other means.

Fig. 4 demonstrates that polymersomes are capable of penetrating deep into the zebrafish tail. Free (R)-roscovitine will likely offer only modest tissue penetration because it has a partition coefficient (logP) of 3.2 and generally only highly hydrophobic molecules are capable of deep tissue penetration; indeed, compounds with a logP below 3.8 have relatively low penetration in zebrafish (74). Delivery of (R)-roscovitine using polymersomes helps improve drug tissue penetration and may offer a more selective uptake into immune cells, compared with other cell types (43, 57, 58). Consequently we believe the superior ability of polymersomes to deliver (R)-roscovitine to neutrophils in the zebrafish tail improved the outcome of this model. Polymersomes may prove an effective vector for the local delivery of drugs to neutrophils.

Neutrophil-dominated inflammatory diseases are common and there are currently no neutrophil-targeted treatments available. In this study we have shown that PMPC-PDPA polymersomes can effectively deliver molecules into human neutrophils without evoking obvious detrimental effects on neutrophil viability or IL-8 release. These polymersomes successfully encapsulated and delivered (R)-roscovitine to promote neutrophil apoptosis in vitro and help resolve a model of neutrophilic inflammation in vivo. These results have important consequences for the study of neutrophil biology, and the development of neutrophil-targeted therapeutics.

## Supplementary Material

Data Supplement
