# Supplementary material for: Targeting Neutrophilic Inflammation Using Polymersome-Mediated Cellular Delivery
Source: J Immunol. 2017 Mar 13;198(9):3596–604. doi: 10.4049/jimmunol.1601901 (PMC5392731; doi:10.4049/jimmunol.1601901)
Supplement: Data Supplement [file JI_1601901.zip › JI_1601901_Supplemental_Figures_1.pdf]

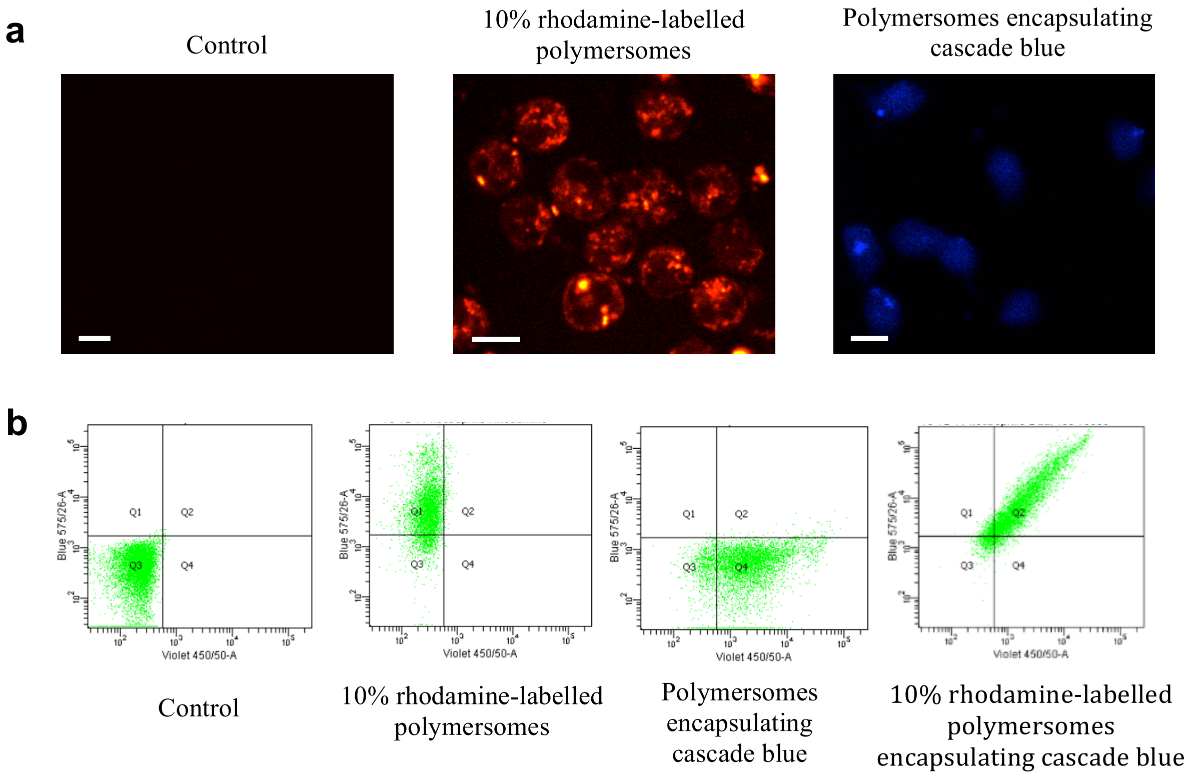

Supplementary Figure 1 Uptake of rhodamine-labelled polymersomes encapsulating cascade blue in human neutrophils.
(a) Confocal micrographs of human neutrophils after incubation with 10% rhodamine-labelled polymersomes (central image) or polymersomes encapsulating cascade blue (right image) for 5 hours. The left image shows a control treated with PBS only. Scale bar = 8μm. (b) Experiment controls showing dot plots for neutrophils without treatment, or neutrophils incubated with polymersomes encapsulating cascade blue, rhodamine labelled polymersomes or rhodamine-labelled polymersomes encapsulating cascade blue.

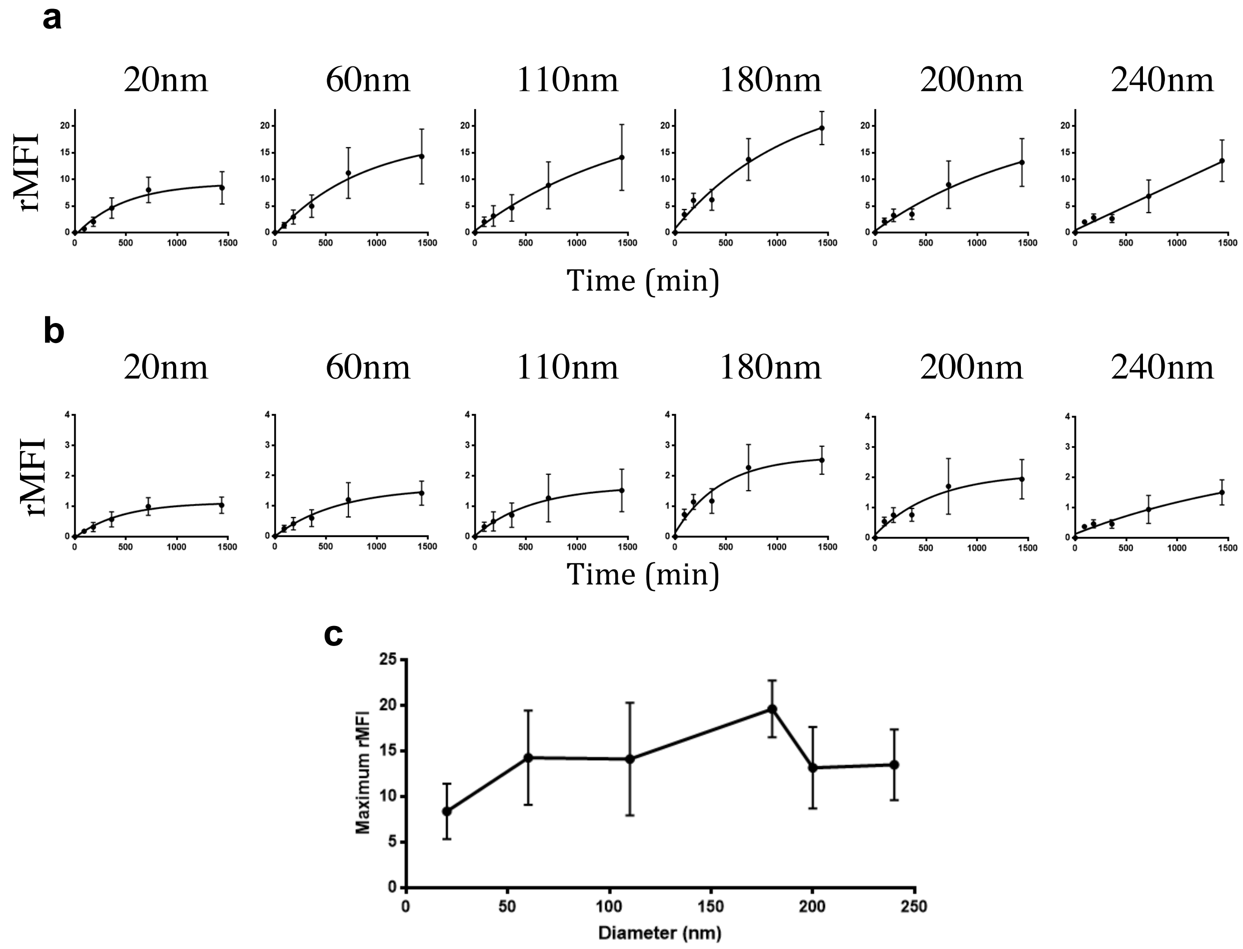

Supplementary Figure 2 Quantified FaDu rMFI over time after incubation with rhodamine labelled polymersomes encapsulating cascade blue.

(a) Fadu rMFI in the rhodamine channel showing the amount of rhodamine-labelled polymer internalised over time for each of the size fractions as measured by flow cytometry. (b) FaDu rMFI in the cascade blue channel showing the amount of cascade blue delivered into FaDu cells over time for each size fraction. (c) FaDu rMFI after incubation with polymersomes at each size fraction for 1500 minutes (on all graphs error bars = SEM, from 3 independent experiments).

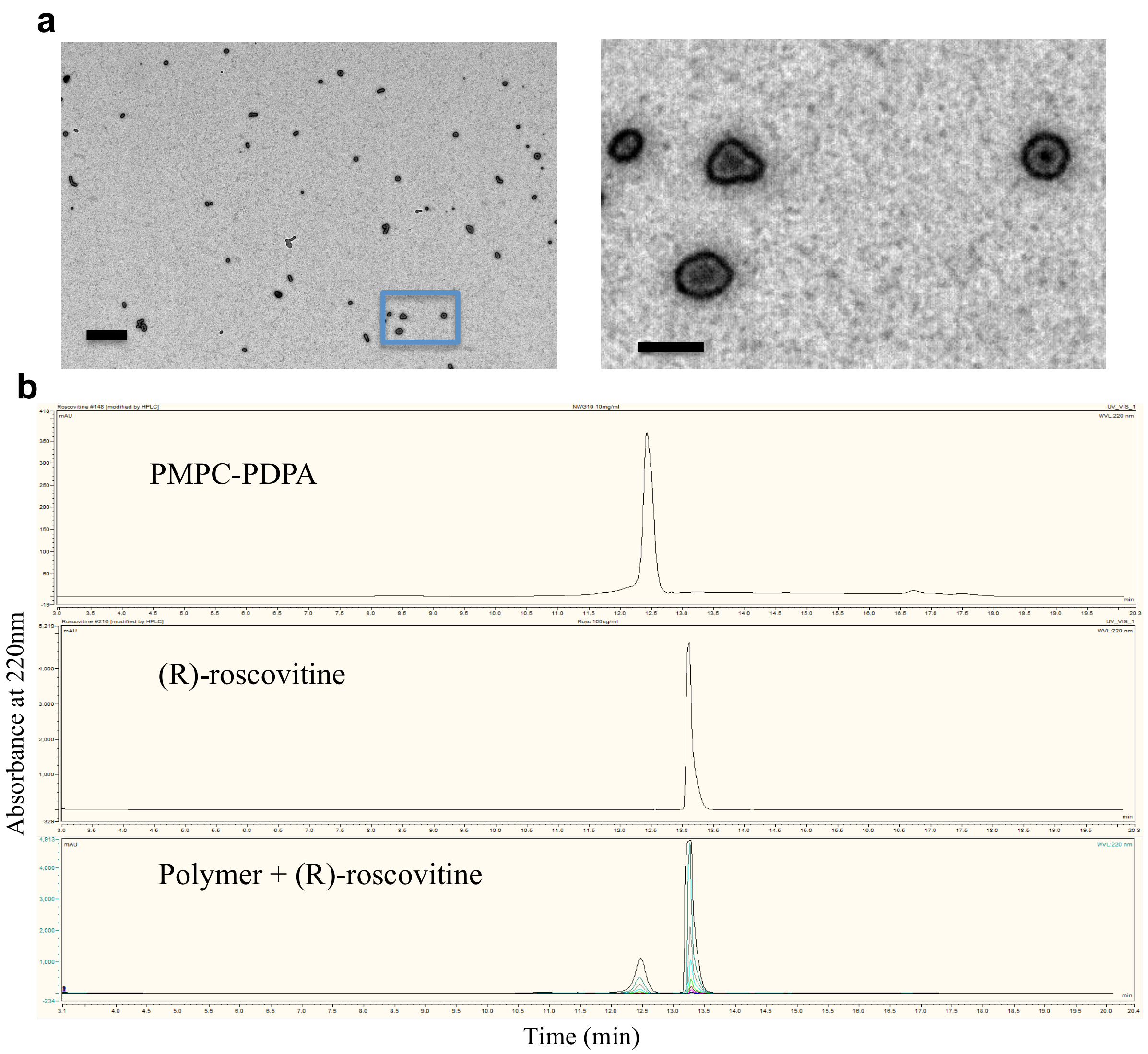

Supplementary Figure 3 Encapsulation of (R)-roscovitine.
(R)-roscovitine was encapsulated by the film rehydration method and polymersomes were purified by differential centrifugation. (a) TEM microscopy of polymersomes encapsulating (R)-roscovitine, scale bar represents 1µm (left image) and 200nm (right magnified image). (b) RP-HPLC chromatograms for the polymer alone (top image) or the (R)-roscovitine alone (central image) or both combined (lower image).
