# Supplementary material for: Targeting Neutrophilic Inflammation Using Polymersome-Mediated Cellular Delivery
Source: J Immunol. 2017 Mar 13;198(9):3596–604. doi: 10.4049/jimmunol.1601901 (PMC5392731; doi:10.4049/jimmunol.1601901)
Supplement: Data Supplement [file JI_1601901.zip › JI_1601901_Supplemental_Material_1.pdf]

Video Legend

Video 1: 3D reconstruction of a single human neutrophil.
3D reconstruction from a confocal z-stack of a human neutrophil after treatment with polymersomes encapsulating rhodamine b octadecyl ester perchlorate.
